# *Filaggrin* null mutations are associated with altered circulating Tregs in atopic dermatitis

**DOI:** 10.1111/jcmm.14031

**Published:** 2018-12-04

**Authors:** Verena Moosbrugger‐Martinz, Robert Gruber, Katharina Ladstätter, Marion Bellutti, Stefan Blunder, Matthias Schmuth, Sandrine Dubrac

**Affiliations:** ^1^ Department of Dermatology, Venereology and Allergology Medical University of Innsbruck Innsbruck Austria

**Keywords:** atopic dermatitis, filaggrin, regulatory *T* cells

## Abstract

Atopic dermatitis (AD) is a chronic inflammatory skin disease with a complex pathogenesis. Although regulatory *T* cells (Tregs) have previously been studied in AD, their role remains controversial, likely owing to patient heterogeneity. Thus, we recruited adult AD patients and age‐matched healthy controls, and assessed their filaggrin (*FLG*) genotype, serum IgE level, and eczema area and severity index (EASI). We found increased proportions of all circulating Treg subpopulations in AD patients. Moreover, we show positive correlations between circulating Tregs and serum IgE *FLG* null mutations limited the expansion of both memory and effector Tregs and enhanced that of recently thymus‐emigrated Tregs. Furthermore, proportions of circulating Th2‐ or Th17‐Tregs but not Th1‐Tregs were increased in AD patients, and accentuated by *FLG* null mutations, thereby mimicking the immune deviation observed in Th cell populations. Moreover, ICOS^+^ Tregs showed reduced production of interleukin‐10, suggesting impaired immunosuppression in AD. The level of demethylation of *FOXP3i1*, which reflects the stability of *FOXP3* expression, was similar in the blood and skin of AD patients and healthy controls. Overall, these results show that Tregs may participate into AD pathogenesis and that *FLG* null mutations exert further modifications on specific subpopulations of circulating Tregs.

## 1 INTRODUCTION

Atopic dermatitis (AD) is a chronic relapsing, pruritic inflammatory skin disease with early onset. It typically presents at around two years of age and then slowly regresses, disappearing before adolescence in 50%‐70% of cases. In rare instances, AD can develop in adults. Worldwide, AD prevalence is relatively high, affecting 15%‐30% of children and 2%‐10% of adults. Notably, AD prevalence has markedly increased during the past 40 years, potentially because of a higher pollution burden.^1^, ^2^, ^3^, ^4^

The pathogenesis of AD is complex and not yet fully understood. Epidermal barrier defects and immune hyper‐responsiveness are the main features of AD. Meta‐analysis of genome‐wide association studies identified a risk locus in the epidermal differentiation complex and especially the *filaggrin* (*FLG*) gene. Furthermore, loss‐of‐function mutations in *FLG* were found to be strong predisposing factors for AD.^5^ Studying immune abnormalities in AD is challenging because of heterogeneity in patient cohorts (eg, children vs adults, high vs normal serum IgE levels, *FLG* wild‐type vs *FLG* mutated), disease course and clinical pattern. However, there is a consensus that AD involves a Th2‐predominant immune response in the acute phase followed by a Th1‐predominant immune response in the chronic phase. Th17, Th22 and Th9 immune responses have been identified in AD as well.^6^, ^7^, ^8^, ^9^, ^10^

Regulatory *T* cells (Tregs) are a subset of CD4^+^ *T* cells involved in immune tolerance. There are two main Treg subpopulations: Tregs emigrated from the thymus and Tregs induced in the periphery by differentiation of naïve conventional *T* cells. Although Tregs dampen inflammation, they are highly plastic and can differentiate into T helper (Th)‐like cells, much like CD4^+^ *T* cells can do. Tregs with the capacity to produce proinflammatory cytokines, such as IL‐17A and IFN‐γ, have been described in cord blood of healthy neonates and in lipopolysaccharide‐induced inflammation.^11^ Moreover, ex vivo restimulation of mouse Tregs in the presence of IL‐4 was found to up‐regulate the *GATA3* gene, which encodes a key transcription factor of the Th2 immune response, whereas the presence of IFN‐γ triggered T‐bet*,* a transcription factor responsible for expansion of Th1 cells.^12^ In mice, Bcl6^−/−^ Tregs are capable of suppressing colitis, but not allergic airway inflammation, and can even exacerbate the Th2 immune deviation by producing IL‐4, IL‐13 and IL‐5 via up‐regulation of *GATA3*.^13^ In contrast, in a mouse model of helminth‐driven inflammation, excess IL‐4 led to expansion of Th2‐like Tregs with preserved suppressive capacities.^14^ Thus, recent literature proves there can be expansion of Tregs producing Th1, Th2 or Th17 cytokines in both the steady‐state and under inflammatory conditions. However, whether these Tregs are still able to dampen ongoing inflammation remains controversial and could depend on the type of inflammation and the microenvironment.

In AD, the percentages of circulating Tregs are consistently found to be increased,^15^, ^16^, ^17^ and this could have both favourable and unfavourable effects. Thymus‐derived Tregs are increased in a mouse model of AD,^18^ similar to other inflammatory disorders.^11^ Interestingly, these cells can redifferentiate into Th‐like cells under inflammatory conditions.^19^ Moreover, restimulation of CCR6^–^ Tregs from AD patients with staphylococcal enterotoxin B enhances their production of IL‐5 and dampens that of IL‐10.^20^ Similarly, restimulation of these cells with CD3 and irradiated monocytes stimulates their production of IL‐5 and IL‐13.^15^ Thus, CCR6^–^ Tregs can be considered as Th2‐like Tregs, as confirmed by previous work,^21^ and promote AD.^15^ Thus, in AD, immunosuppressive Tregs might cohabit with redifferentiated Tregs able to produce pro‐inflammatory mediators. Therefore, it is not surprising that the role of Tregs in AD remains controversial. Moreover, the broad heterogeneity of AD patients and the inclusion of patients under various therapies in some studies further complicate the results.^22^

The discovery that Tregs have a role in cancer has aided in the development of therapeutic antibodies to anti‐cytotoxic T lymphocyte associated antigen 4 (CTLA4) and anti‐programmed cell death protein 1 (PD1) as a novel and promising immunotherapeutic approach.^23^ If the role of Tregs can be elucidated in AD, then the use of such therapies targeting Treg function might provide a valuable and complementary approach to AD treatment. Ideally, it would be best to uncover the role(s) of Tregs in all AD patient subpopulations. Towards that goal, we recruited adult patients not currently undergoing therapy with similar disease stages (EASI = 3.5 ± 1.0, n = 13) and known serum IgE levels, and whom we genotyped for *FLG* null mutations. We found that *FLG* null mutations target specific aspects of Tregs in AD.

## 2 MATERIALS AND METHODS

### 2.1 Human subjects

The study was approved by the Ethics Committee of the Medical University of Innsbruck and conducted in accordance with the Declaration of Helsinki principles. All study subjects gave written informed consent and participated voluntarily. All patients and healthy controls are of Non‐Finnish European origin. Blood was taken in EDTA tubes. All study subjects (including controls) were screened for *FLG* mutations by sequencing exon 3 of the filaggrin gene. The study included both AD patients without a *FLG* mutation (AD WT/WT) and AD patients heterozygous for a *FLG* mutation (AD *FLG* MUT), as well as healthy control subjects without a *FLG* mutation (CTRL). All AD *FLG* MUT patients were heterozygous for c.2282_2285delCAGT, except two patients who were heterozygous for c.3418C>T and c.2513_2525dupAGGACACCATTCG respectively. Patient characteristics are summarized in Tables S1 and S2. All AD patients had a disease history since childhood. None of the patients had used an emollient or any other topical formulation for at least 5 days prior to study participation. Patients treated with phototherapy or a systemic immunosuppressant drug were excluded from the study. Skin biopsies were taken from non‐UV‐irradiated trunk skin of another cohort of AD patients (AD) with and without a *FLG* mutation and healthy control subjects. In this second cohort, AD patients with *FLG* mutations were either compound‐heterozygous for c.2282_2285delCAGT and c.7339C>T, or heterozygous for c.1501C>T. Skin erythema was measured with a Multi Probe Adapter System (MP6, Courage & Khazaka, Köln, Germany).

### 2.2 Antibodies

Directly labelled primary monoclonal human antibodies specific for CD4, CD25, CD127, CCR4, ICOS, CD161, CXCR3, CCR6, CD45RA and CD31, as well as biotinylated anti‐human IL‐10 and corresponding isotype controls and streptavidin PerCP‐Cy5.5 were purchased from BioLegend (San Diego, CA). Live‐dead was purchased from Life Technologies (Carlsbad, CA) and Zombie green^TM^ from BioLegend (San Diego, CA).

### 2.3 Flow cytometry analysis

Blood cells were either directly used for flow cytometry analysis or restimulated in vitro with 2 μg/mL coated antiCD3 and 4 μg/mL soluble antiCD28 (BD Pharmingen, San Diego, CA) for 24 hours at 37°C. For intracellular cytokine production, cytokine release was blocked with 1 μg/mL Brefeldin A (BD Biosciences, San Diego, CA) during the last 4 hours of restimulation. All stainings for surface molecules were performed for 15 minutes at 4°C. Cell fixation prior to cytokine staining was done using a BD Biosciences Cytofix/Cytoperm Kit according to the manufacturer's protocol for 30 minutes at room temperature. Dead cells were excluded by Live‐dead or Zombie green staining. FACS analyses were performed on a FACS Canto II instrument (BD Biosciences). Data analysis was performed withClick here to enter text. FlowJo software (Tree Star, Ashland, OR). Cell frequencies in lymphocyte populations were calculated as follows: (% positive cells × % Tregs × % CD4 ^+^ *T* cells)/10 000.

### 2.4 Demethylation of intron 1 of the *FOXP3* gene

Genomic DNA (gDNA) was extracted from peripheral blood as previously described.^24^ 500 ng of gDNA was treated with sodium bisulfite using the MethylCodeBisulfite Conversion kit (Invitrogen, Carlsbad, CA). Real‐time PCR amplifications for quantification of methylated and unmethylated *FoxP3i1* sequences were performed on 50 ng of bisulfite‐converted DNA in a final volume of 30 μl with 300 nM of each primer, 100 nM of probe and TaqMan Brilliant III Ultra Fast QPCR Master Mix from Agilent Technologies (Santa Clara, CA) using a CFX96 Touch Real‐Time PCR Detection System (Bio‐Rad, Hercules, CA). PCR conditions were 94°C for 10 minutes, 45 cycles of 94°C for 15 seconds and 64°C for 1 minute. Sequences of primers and probes were as indicated in Stockis et  al^25^ and de Vries et  al.^26^ Proportions of cells with unmethylated *FoxP3i1* were calculated as previously described.^26^

### 2.5 Statistical analysis

Statistical analyses were performed with GraphPad Prism 6 software (GraphPad Software, La Jolla, CA). Data are presented as mean ± SEM. Statistical significance was determined between groups using a Student′s *t* test or one‐way ANOVA followed by a Tukey post‐hoc test with *P < 0.05, **P < 0.01, ***P < 0.0001.

## 3 RESULTS

### 3.1 Disease severity but not *FLG* null mutations correlates with expansion of circulating Tregs in AD

**Figure 1:**
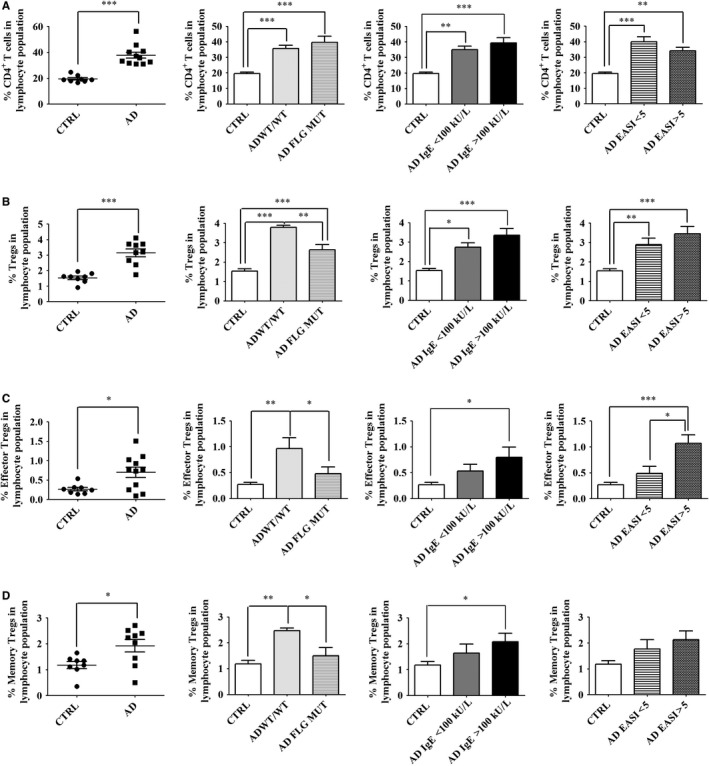
*FLG* null‐mutations limit the expansion of circulating effector and memory Tregs in AD. Freshly collected blood cells were analysed by flow cytometry. Live lymphocytes were gated on CD4. Tregs were designated as CD4^+^CD25^+^CD127^low/–^ cells as depicted in Figure S1, effector (CD45RA^–^CCR4^+^) Tregs as depicted in Figure S2, and memory (CD45RA^–^ICOS^+/–^CD31^+/–^) Tregs as depicted in Figure S3. Results show cell frequencies in the lymphocyte population as calculated in Materials and Methods. Percentages of (A) CD4^+^ cells, (B) Tregs, (C) effector Tregs and (D) memory Tregs. Data were analysed using a Student′s *t* test or one‐way ANOVA followed by a Tukey post‐hoc test with *P < 0.05, **P < 0.01, ***P < 0.0001. Healthy controls are designated as CTRL (n = 8); AD patients as AD (n = 11), including patients without a *FLG* null mutation as AD WT/WT (n = 5) and patients with a *FLG* null mutation as AD *FLG* MUT (n = 6). AD patients were also stratified according to serum IgE level (<100 kUI/l, n = 4; >100 kUI/l, n = 7) or EASI score (<5, n = 7; >5, n = 4)

The literature on Tregs in AD is controversial because of patient heterogeneity in many studies. Thus, to circumvent this confounder, we recruited adult AD patients and healthy controls close in age (35.3 ± 2.6 years old, n = 23, Table S1). All patients and healthy controls were examined for *FLG* loss‐of‐function mutations, serum IgE level, and eczema area and severity index (EASI) (Table S1). Other participant characteristics are summarized in Tables S1 and S2. In our cohort, EASI score and serum IgE level were similar among AD patients, regardless of *FLG* genotype (Table S1). In this cohort, *FLG* mutated AD patients did not exhibit a more severe disease phenotype than *FLG* wild‐type AD patients, in accordance with some studies but in contrast to others.^27^, ^28^ We first assessed the proportion of Tregs in the total lymphocyte population, because lymphocyte population size is more stable than CD4^+^ *T* cell population size (Table S2, Figure 1A). Indeed, the percentage of CD4^+^ *T* cells was higher in the blood of AD patients when compared to healthy controls, regardless of *FLG* mutation status, serum IgE level and EASI score (Figure 1A). In contrast, the size of the lymphocyte population remained similar in AD patients and healthy controls (Table S2 and data not shown). Thus, to avoid bias due to changes in population size, we expressed all results as percentages of cells in the lymphocyte population (see calculation in Materials and Methods). Moreover, this calculation better reflects the changes in circulating populations caused by the pathology. The gating strategy for Tregs is depicted in Figure S1. We found that the proportion of circulating CD4^+^CD25^+^CD127^low/–^ cells, considered as Tregs, was increased in AD patients when compared to healthy controls (Figure 1B). Furthermore, higher serum IgE levels and EASI scores were found to be associated with higher levels of circulating Tregs in AD patients, in contrast to the non‐influence we observed for *FLG* null mutations (Figure 1B). We also found a positive correlation between the percentage of circulating Tregs and serum IgE levels (*r* ^2^ = 0.4585, *P* = 0.0028, n = 17) and skin erythema (*r* ^2^ = 0.6087, *P* = 0.0002, n = 17). All together, these results suggest a tight relationship between circulating Tregs and AD symptoms.

### 3.2 *FLG* null mutations limit the expansion of circulating effector and memory Tregs in AD

We next characterized the population of circulating Tregs in the blood of AD patients and healthy control subjects. We used CCR4 and CD45RA expression to measure the levels of circulating effector (CD45RA^–^CCR4^+^) Tregs, as depicted in Figure S2.^29^ We also designated memory Tregs as CD4^+^CD25^+^CD127^low/–^CD45RAICOS^+/–^CD31^+/–^ cells, as depicted in Figure S3.^30^ We found that the proportions of effector and memory Tregs were increased in the blood of AD patients (Figure 1C‐D) and followed similar modulations as the whole Treg population (Figure 1B). Thus, AD is characterized by expansion of circulating effector Tregs, potentially in response to exposure to foreign antigens,^31^, ^32^ and conversion of effector Tregs into memory cells is not impaired. Moreover, the dichotomy between ICOS^–^ and ICOS^+^ memory Tregs shows that only ICOS^+^ memory Tregs are significantly increased (Figure S4). Thus, in AD, Treg expansion appears to be due mainly to increased expansion of memory Tregs (Figure 1B‐D) and limited by *FLG* null mutations.

### 3.3 *FLG* null mutations are associated with expansion of thymus‐emigrated Tregs in AD

**Figure 2:**
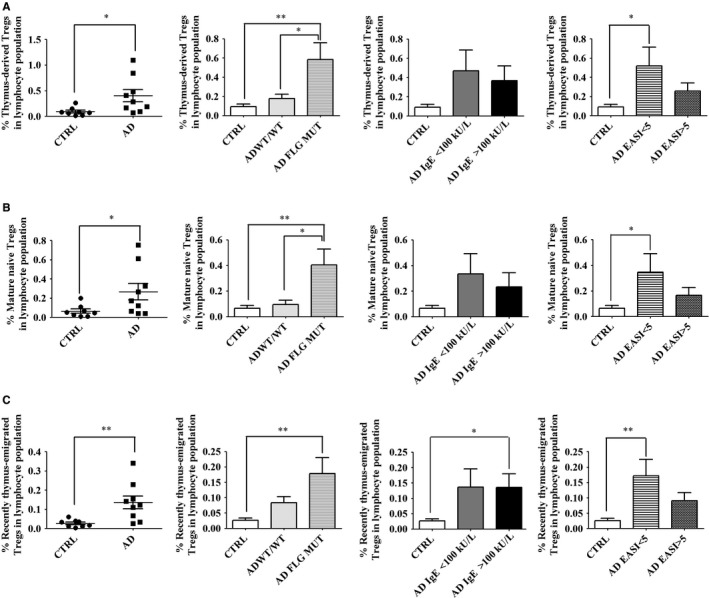
*FLG* null mutations are associated with expansion of thymus‐emigrated Tregs in AD. Freshly collected blood cells were analysed by flow cytometry. Live lymphocytes were gated on CD4. Tregs were designated as CD4^+^CD25^+^CD127^low/–^ cells as depicted in Figure S1, thymus‐derived (CD45RA^+^CD31^+/–^) Tregs, mature naïve (CD45RA^+^CD31^–^) Tregs, and recently thymus‐emigrated (CD45RA^+^CD31^+^) Tregs as depicted in Figure S3. Results show cell frequencies in the lymphocyte population as calculated in Materials and Methods. Percentages of (A) thymus‐derived, (B) mature naïve and (C) recently thymus‐emigrated Tregs. Data were analysed using a Student′s *t* test or one‐way ANOVA followed by a Tukey post‐hoc test with *P < 0.05, **P < 0.01. Healthy controls are designated as CTRL (n = 8); AD patients as AD (n = 11), including patients without a *FLG* null mutation as AD WT/WT (n = 5) and patients with a *FLG* null mutation as AD *FLG* MUT (n = 6). AD patients were also stratified according to serum IgE level (<100 kU/l, n = 4; >100 kU/l, n = 7) or EASI score (<5, n = 7; >5, n = 4)

Recently, we showed that thymus‐derived Tregs are augmented in a mouse model of AD, induced by topical application of vitamin D3.^18^ Here, we assessed if this also holds true for patients with AD. We gated thymus‐derived Tregs as depicted in Figure S3.^30^ We found the proportion of thymus‐derived Tregs to be significantly greater in AD patients with *FLG* null mutations compared to both healthy donors and AD patients with wild‐type *FLG* (Figure 2A). Thymus‐derived Tregs can be further split into mature naïve (CD4^+^CD25^+^CD127^low/–^CD45RA^+^CD31^–^) and recently thymus‐emigrated (CD4^+^CD25^+^CD127^low/–^CD45RA^+^CD31^+^) Tregs, as depicted in Figure S3.^30^ Enhanced expansion of mature naïve Tregs was observed only in AD patients bearing *FLG* null mutations (Figure 2B),^33^ similar to the proportions of circulating recently thymus‐emigrated Tregs (Figure 2C). Expansion of mature naïve Tregs resulted from expansion of ICOS^+^ mature naïve Tregs (Figure S4), whereas the increased proportion of recently thymus‐emigrated Tregs resulted from expansion of both the ICOS^+^ and ICOS^–^ fractions (Figure S5). Hence, these results show enhanced levels of thymus‐emigrated Tregs specifically in blood of AD patients with *FLG* null mutations.

### 3.4 Proportions of cells with demethylated *FOXP3i1* are not altered in the blood of patients with AD

**Figure 3:**
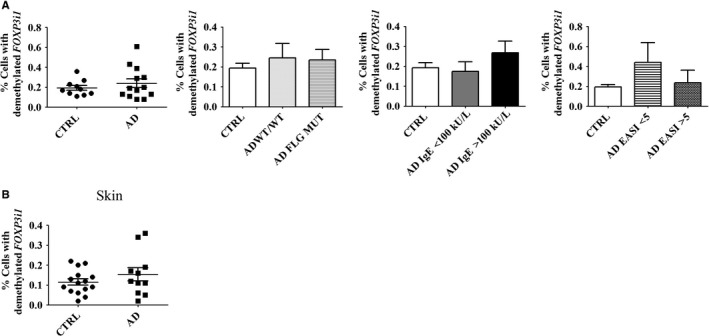
Proportions of cells with demethylated *FOXP3i1* are not altered in the blood of patients with AD. Proportions of cells with demethylated *FOXP3i1* (A) in the blood or (B) in the skin of healthy controls and AD patients. Data were analysed using a Student′s *t* test or one‐way ANOVA followed by a Tukey post‐hoc test. Healthy controls are designated as CTRL (blood, n = 10; skin, n = 15); AD patients as AD (blood, n = 13; skin, n = 11), including patients without a *FLG* null mutation as AD WT/WT (blood, n = 7; skin, n = 7) and patients with a *FLG* null mutation as AD *FLG* MUT (blood, n = 6; skin, n = 4). AD patients were also stratified according to serum IgE level (<100 kU/l, blood, n = 4; >100 kU/l, blood, n = 9) or EASI score (<5, blood, n = 9; >5, blood, n = 4)

The literature is contradictory on the immunosuppressive capacity of Tregs in AD. One study concluded that the autoimmune reaction in severe AD is due to an attenuated Treg suppressive function,^34^ whereas another study found no difference in Treg immunosuppressive capacity in AD patients and healthy donors.^15^ Moreover, bulk Tregs isolated from AD patient blood are capable of suppressing autologous effector *T* cells.^15^ Demethylation of intron 1 of *FOXP3* (*FOXP3i1*) is known to stabilize expression of the gene in Tregs and sustain their immunosuppressive capacity.^35^ We found no difference in the proportion of Tregs with demethylated *FOXP3i1* in the blood of AD patients and healthy controls, regardless of *FLG* null mutation status, serum IgE level and EASI score (Figure 3A). We found similar results in the skin (Figure 3B and data not shown). Thus, in AD, the stability of *FOXP3* expression in circulating Tregs is not altered.

### 3.5 Production of IL‐10 by ICOS^+^ Tregs is impaired by cell death in AD

**Figure 4:**
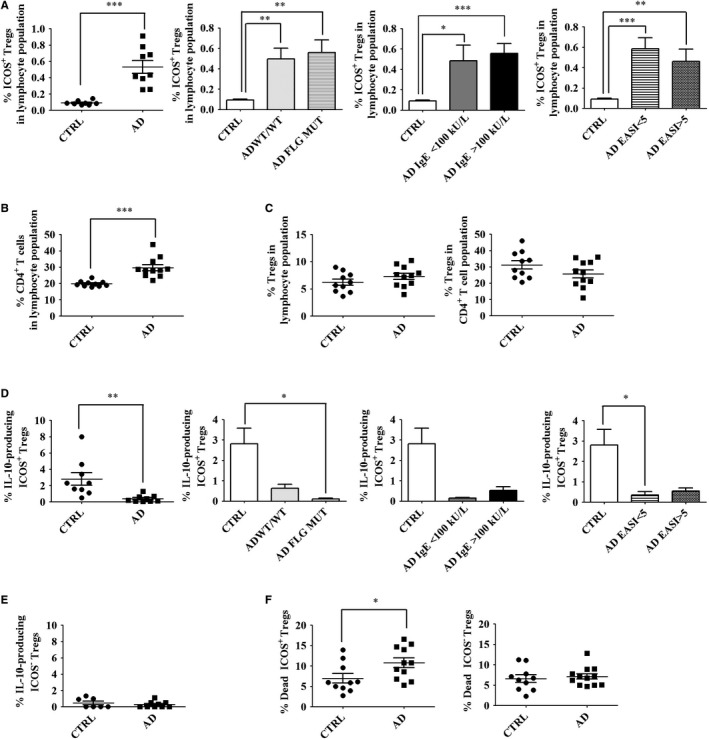
Production of IL‐10 by ICOS^+^ Tregs is impaired by cell death in AD. Freshly collected blood cells were restimulated by antiCD3/antiCD28 for 24 h and then analysed by flow cytometry as described in Materials and Methods. Live lymphocytes were gated on CD4. Tregs were designated as CD4^+^CD25^+^CD127^low/–^ cells as depicted in Figure S1 and further stratified by ICOS expression. A, Percentages of ICOS^+^ Tregs in the lymphocyte population as calculated in Materials and Methods. B, Percentages of CD4^+^ *T* cells after restimulation in the lymphocyte population, (C) Percentages of Tregs after restimulation in the lymphocyte (left panel) or in the CD4^+^ *T* cell population (right panel). Percentages of IL‐10‐producing (D) ICOS^+^ Tregs or (E) ICOS^–^ Tregs. (F) Percentages of apoptotic/necrotic ICOS^+^ Tregs (left panel) or ICOS^–^ Tregs (right panel). Data were analysed using a Student′s *t* test or one‐way ANOVA followed by a Tukey post‐hoc test with *P < 0.05, **P < 0.01, ***P < 0.0001. Healthy controls are designated as CTRL (n = 9); AD patients as AD (n = 9), including patients without a *FLG* null mutation as AD WT/WT (n = 5) and patients with a *FLG* null mutation as AD *FLG* MUT (n = 4). AD patients were also stratified according to serum IgE level (<100 kU/l, n = 3; >100 kU/l, n = 6) or EASI score (<5, n = 6; >5, n = 3)

Effector/memory Tregs express high levels of ICOS, which contributes to the survival and expansion of Tregs.^36^ Moreover, ICOS^+^ Tregs, which likely derive from expansion of thymus‐derived Tregs, are potentially highly suppressive.^37^ We found that the percentages of ICOS^+^ Tregs are increased in the blood of AD patients, regardless of *FLG* mutation status, serum IgE level and EASI score (Figure 4A). All Treg populations produce IL‐10, whose proper secretion is necessary for immunosuppression.^38^ However, ICOS^+^ Tregs produce higher amounts of IL‐10 compared to ICOS^–^ Tregs and other *T* cells.^37^ Moreover, ICOS is believed to be part of a switch mechanism for the production of IL‐10.^39^ To measure IL‐10 production by Tregs, we restimulated cells with antiCD3/antiCD28 for 24 hours. First, we measured the percentages of CD4^+^ and Tregs after in vitro restimulation to determine if restimulation might alter the size of lymphocyte populations. We found that the percentages of CD4^+^ *T* cells were still increased in AD‐derived cell cultures compared to those derived from healthy controls (Figure 4B). However, the proportions of Tregs in the lymphocyte population were similar in AD and control cultures (Figure 4C), in contrast to whole blood (Figure 1B), hence suggesting Treg loss upon restimulation in AD. Then, we measured the production of IL‐10 by Tregs stratified by ICOS expression. Note that, the proportions of ICOS^+^ Tregs were similar in both the patient and control groups after restimulation (data not shown). We observed a decreased capacity of ICOS^+^ Tregs from AD patients to produce IL‐10 when compared to Tregs from healthy controls, regardless of *FLG* genotype and disease severity (Figure 4D). ICOS^–^ Tregs in both groups produced very little IL‐10 (Figure 4E), as reported previously.^39^ Impaired IL‐10 production by ICOS^+^ Tregs could result from defective IL‐10‐biosynthetic machinery or reduced cell vitality. We found ICOS^+^ Tregs from AD patients to be more susceptible to cell death upon restimulation when compared to cells derived from healthy controls (Figure 4F). In contrast, both groups presented similar numbers of dead ICOS^–^ Tregs (Figure 4F). Thus, our results suggest that, despite increased frequencies of circulating ICOS^+^ Tregs in AD, their efficacy of immunosuppression might be impaired due to reduced viability upon restimulation which would, in turn, lead to impaired production of IL‐10. Moreover, the susceptibility of ICOS^+^ Tregs to cell death after restimulation might be independent of the stability of *FOXP3* expression (Figure 3A).

### 3.6 *FLG* null mutations amplify immune imbalance in Treg populations in AD patients

**Figure 5:**
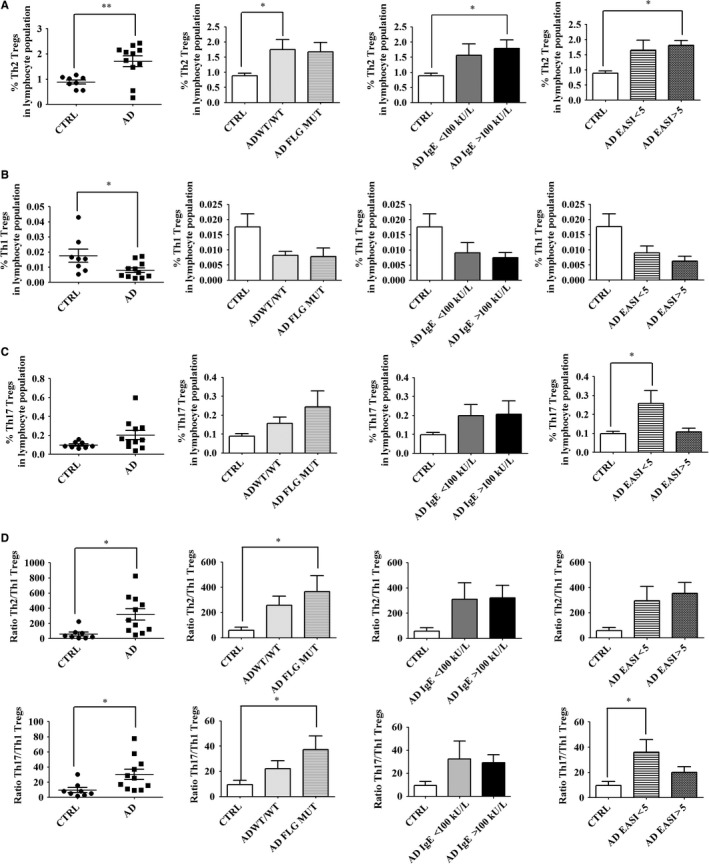
*FLG* null mutations amplify immune imbalance in Treg populations in patients with AD. Freshly collected blood cells were analysed by flow cytometry. Live lymphocytes were gated on CD4. Tregs were designated as CD4^+^CD25^+^CD127^low/–^ cells as depicted in Figure S1. Th1‐, Th2‐ and Th17 ‐like Tregs were designated as CCR4^–^CXCR3^+^, CCR4^+^CXCR3^–^CCR6^–^ and CCR4^+^CXCR3^–^CCR6^+^CD161^+^ respectively. Results show cell frequencies in the lymphocyte population as calculated in Materials and Methods. Percentages of (A) Th2‐, (B) Th1‐ and (C) Th17‐like Tregs. (D) Ratio between frequencies of Th2‐ versus. Th1‐like Tregs (upper panel) and between Th17‐ versus Th1‐like Tregs (lower panel). Data were analysed using a Student′s *t* test or one‐way ANOVA followed by a Tukey post‐hoc test with *P < 0.05, **P < 0.01. Healthy controls are designated as CTRL (n = 8); AD patients as AD (n = 11), including patients without a *FLG* null mutation as AD WT/WT (n = 5) and patients with a *FLG* null mutation as AD *FLG* MUT (n = 6). AD patients were also stratified according to serum IgE level (<100 kU/l, n = 4; >100 kU/l, n = 7) or EASI score (<5, n = 7; >5, n = 4)

It is well‐established that Tregs are as plastic as *T* cells. They can be skewed towards Th1‐, Th2‐ or Th17‐like cells via up‐regulation of T‐bet, GATA3 or RORγ respectively.^12^, ^21^, ^40^ Th1 (CCR4^–^CXCR3^+^)‐, Th2 (CCR4^+^CXCR3^–^CCR6^–^)‐ and Th17 (CCR4^+^CXCR3^–^CCR6^+^CD161^+^)‐like Tregs have been previously identified via expression of surface markers,^21^, ^41^ similar to Th cells.^42^ Thus, using the same strategy, we assessed the proportions of Th1‐, Th2‐ and Th17‐like Tregs in the blood of all participants. We first showed that Th1‐, Th2‐ and Th17‐like cells are present in the blood of healthy donors, with a predominance of Th2‐like cells (Figure S6). We found that the proportions of Th2‐like Tregs were significantly increased in the blood of AD patients when compared to healthy controls, regardless of *FLG* mutation status, serum IgE level and EASI score, whereas the proportions of Th1‐like Tregs were significantly reduced (Figure 5A‐B and Figure S6). In the case of Th17‐like Tregs, they showed a tendency to be elevated in AD, especially in patients with a *FLG* null mutation (Figure 5C). Furthermore, ratios show a Th2/Th1 and Th17/Th1 immune imbalance in AD patients, especially in those with a *FLG* null mutation (Figure 5D). There is no Th2/Th17 imbalance in AD patients when compared to healthy donors (data not shown). Thus, in AD patients, circulating Tregs might be identically skewed towards a predominant Th2/Th17 immunity as observed in Th cell populations, thereby potentially amplifying immune abnormalities, especially in patients with *FLG* null mutations.

## 4 DISCUSSION

AD is one of the most common skin diseases worldwide, with high comorbidity in its more severe forms. Indeed, AD can be accompanied by psychosocial problems, sleep deprivation, anorexia, depression and even suicidal ideation.^39^ Unfortunately for the afflicted, there is no cure for AD and current therapeutic approaches are symptomatic despite extensive research.^9^ Thus, identification of new potential therapeutic targets remains of interest for AD patients. The discovery of the key role of Tregs in cancer has spurred the development of new therapies based on checkpoint blockade.^23^ However, in AD, a definitive role of Tregs remains to be demonstrated, thus, precluding Treg therapeutic targeting. Nonetheless, a consensus is emerging in support of elevated circulating Tregs in AD.^15^, ^16^, ^17^, ^33^, ^43^, ^44^, ^45^ Moreover, up‐regulation of activation markers on circulating Tregs in adult AD is also well documented.^22^ In contrast, in paediatric AD, there are fewer studies and the findings are more controversial.^46^, ^47^ Contradictory results may be explained, in part, by the effects different treatments (eg, cyclosporine, glucocorticoids, UV irradiation) have on Tregs.^17^ Thus, in this study, to circumvent age‐ and treatment‐related bias, we recruited exclusively adult patients with no history of AD therapy. We found the levels of circulating Tregs to be increased in these patients, potentially due to expansion of memory Tregs, especially the ICOS^+^ fraction, but that *FLG* null mutations limit this expansion.

Thymus‐derived Tregs maintain central immune tolerance and are generated in response to intermediate/high‐affinity interactions with self‐antigens,^48^, ^49^ as well as after UVB exposure.^50^ Elevated numbers of thymus‐derived Tregs have been detected in the circulation of AD patients^51^ and in the skin‐draining lymph nodes of mice exhibiting AD‐like symptoms.^18^ Here, we showed that the proportion of circulating thymus‐derived Tregs is higher in AD patients heterozygous for a *FLG* null mutation than in AD patients without a *FLG* null mutation. FLG has been detected in both mouse and human thymic medulla and FLG deficiency is associated with expansion of IL‐17A‐producing γ/δ *T* cells.^52^, ^53^ Thus, due to its thymic expression, FLG deficiency in AD might affect not only skin, but also various populations of circulating lymphocytes. Interestingly, the Th2 cytokine IL‐4, in the presence of antigen, is able to promote the development of thymus‐derived Tregs.^54^ Thus, increased penetration of antigens through the skin, owing to *FLG* null mutations, might synergize with the immune environment to trigger the expansion of thymus‐derived Tregs in AD patients.

Thymus‐derived Tregs are stable under homoeostatic conditions but can differentiate into Th‐like Tregs under inflammatory conditions.^19^ Yet, despite a fully demethylated Treg‐specific demethylated region (TSDR), thymus‐derived Tregs can re‐differentiate into Th cells and express proinflammatory cytokines, especially in inflammatory diseases that they might, in turn, exacerbate. Thus, although in vitro studies have shown an immunosuppressive capacity of thymus‐derived Tregs,^54^ it remains uncertain whether these Tregs are effective at dampening inflammation in AD. The microenvironment plays a preponderant role in the redifferentiation of thymus‐derived Tregs, with IL‐6, for example, promoting their conversion into Th17‐like Tregs.^55^, ^56^, ^57^ Moreover, recent findings indicate that Treg conversion into Th2‐like cells might exacerbate the Th2 immune response and limit Treg cell‐mediated immunosuppression in oral allergy and allergic airway inflammation.^13^, ^58^ Furthermore, in a previous work, we showed increased proportions of Tregs producing Th2 cytokines in a mouse model of AD.^18^ Here, we showed that Th2/Th1 and Th17/Th1 immune imbalance in AD patients is not restricted to *T* cells, but extends to Tregs and is further triggered by *FLG* null mutations. Thus, in AD, Tregs may sustain inflammation instead of alleviating it. This is supported by recent data showing that partial or complete thymectomy in children significantly reduces the risk of developing AD^59^ but in contrast to other data showing no effect of thymectomy on AD incidence.^60^ Thus, further studies, with stringent technical methods and algorithms that are more complex, are required to clarify the role of thymus‐derived lymphocytes in AD. Interestingly, phenanthrene, a polycyclic aromatic hydrocarbon and a major constituent of urban air pollution, has been shown to promote methylation of the TSDR and convert Tregs into Th2‐like cells. Tregs exposed to phenanthrene show decreased TGF‐β and IL‐10 and increased IL‐4, IL‐13, (p)STAT6 and GATA‐3.^61^ It has recently been shown that repeated exposure to air pollution and to noxious molecules may promote AD.^1^, ^2^, ^3^, ^4^ Thus, pollutants may contribute to AD also via their ability to affect Treg populations, especially by impeding their immunosuppressive capacity by causing them to redifferentiate into Th‐like cells that produce proinflammatory cytokines.

Increased frequencies of ICOS^+^ Tregs have previously been found in the skin of AD patients.^62^ Most Tregs express ICOS, which promotes their survival and expansion.^36^ We found increased frequencies of circulating ICOS^+^ Tregs in AD patients versus healthy controls, regardless of *FLG* mutation status or disease severity. ICOS^+^ Tregs preferentially produce IL‐10, whereas ICOS^–^ Tregs use TGF‐β.^37^ Indeed, ICOS is believed to be part of a switch mechanism for IL‐10 production in Tregs.^39^ Here, we found impaired production of IL‐10 in the main immunosuppressive Treg population, that is, ICOS^+^ Tregs in AD patients, despite similar levels of cells with demethylated *FOXP3i1*.^35^, ^63^ These results can be explained by the observation of increased ICOS^+^ Treg cell death after ex vivo restimulation. Moreover, stabilization of *FOXP3* expression via low gene methylation can only impact the immunosuppressive capacity of cells which remain fit. This suggests that the stability of *FOXP3* expression in Tregs is not linked to and does not promote cell survival.^64^ Furthermore, expression of ICOS by Tregs can generate instability and reprogramming through the PI3K/AKT pathway, which, in turn, is detrimental to their suppression activity.^19^ Thus, ICOS^+^ Treg viability may be impaired upon restimulation in patients with AD, hence impeding their production of IL‐10 and, in turn, the efficacy of their immunosuppression.

In conclusion, we showed that in AD patients, *FLG* null mutations enhance the levels of circulating thymus‐derived Tregs and limit the expansion of effector/memory Tregs, thereby further affecting the Treg landscape in AD patients. Furthermore, *FLG* null mutations aggravate the immune imbalance between circulating Th2‐, Th1‐ and Th17‐like Tregs in AD. IL‐10 production by ICOS^+^ Tregs is abolished in AD patients, potentially due to impaired cell survival after restimulation, suggesting that the levels of demethylation of *FOXP3i1* observed in these cells are not sufficient to sustain their immunosuppressive function. Hence, Treg viability should be thoroughly evaluated. Thus, circulating Tregs in AD patients exhibit some phenotypic alterations that could significantly contribute to AD pathogenesis. Furthermore, this work shows that *FLG* null mutations further promote Treg abnormalities in AD. Developing and testing the effects of drugs which can limit the expansion of Tregs and/or their conversion into Th‐like cells would be worthwhile next steps in the therapeutic approach to AD.

## CONFLICT OF INTEREST

The authors confirm that there are no conflicts of interest.
