# Supplementary material for: Filaggrin null mutations are associated with altered circulating Tregs in atopic dermatitis
Source: J Cell Mol Med. 2018 Dec 4;23(2):1288–99. doi: 10.1111/jcmm.14031 (PMC6349342; doi:10.1111/jcmm.14031)
Supplement: Supplementary file 1 [file JCMM-23-1288-s001.docx]

**Table S1: Characteristics of patient cohort**

| Groups | *FLG* genotype | N | Mean age  ±  SEM | Mean EASI  ±  SEM | Mean total serum IgE  kU/l  ±  SEM | % patients with coexisting allergic disease |
| --- | --- | --- | --- | --- | --- | --- |
| CTRL | wildtype | 10 | 34.8±4.0 | n.s. | 63±28 | n.a. |
| AD WT/WT  AD *FLG* MUT | wildtype  heterozygote | 7  6 | 36.6±4.9  34.5±6.1 | 4.5±1.7  2.4±0.6 | 1297±506  1686±1526 | 71.4  100 |

Healthy controls are designated as CTRL, patients with AD without *FLG* null mutation as AD WT/WT, AD patients with *FLG* null mutation as AD *FLG* MUT, n.s.: not scored, n.a.: not assessed. Statistical significance was determined between groups using a one-way ANOVA test followed by a Tukey post-hoc test.

**Table S2: Blood cells in patient cohort.**

| Groups | % Leukocytes | % Segmented  neutrophils | % Lymphocytes | % Eosinophils | %  Basophils |
| --- | --- | --- | --- | --- | --- |
| CTRL | 7.1±0.5 | 61.0±2.7 | 30.1±2.4 | 1.7±0.4 | 0.6±0.1 |
| AD WT/WT  AD *FLG* MUT | 7.8±0.7  6.4±0.9 | 66.5±4.0  58.2±4.9 | 22.4±3.0  29.6±4.0 | 3.3±1.0  3.8±1.0 | 0.5±0.1  0.6±0.1 |

Healthy controls are designed as CTRL, patients with AD without *FLG* null mutations as AD WT/WT, AD patients with *FLG* null mutations as AD *FLG* MUT. Statistical significance was determined between groups using a one-way ANOVA test followed by a Tukey post-hoc test.

**Fig S1: Gating strategy for Tregs**

Blood cells were gated on size and granularity, then alive (Live-dead^-^ or Zombie green^-^) CD4^+^ cells were further gated. Tregs were designated as CD4^+^CD25^+^CD127^low/-^ cells. Isotype controls were used to define gates.


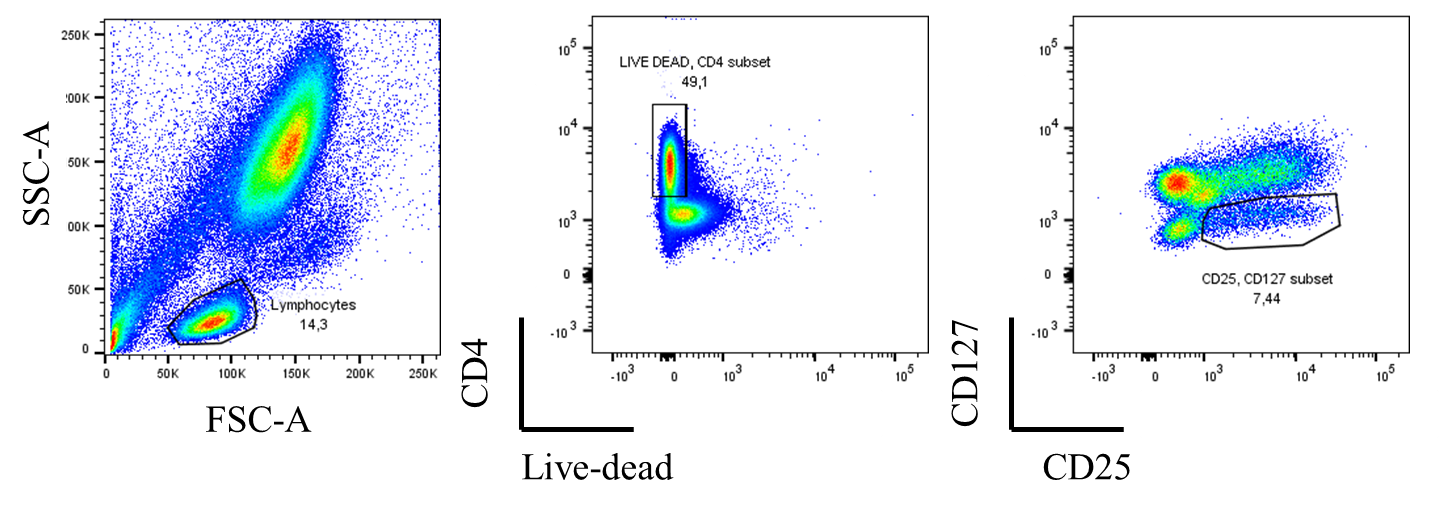


**Fig S2: Gating strategy to define Treg subsets**

Tregs were gated as CD4^+^CD25^+^CD127^low/-^ cells as described in Fig S1. Naïve Tregs were further designated as CD45RA^+^CCR4^-^ cells and effector Tregs as CD45RA^-^CCR4^+^ cells. Isotype controls were used to define gates.


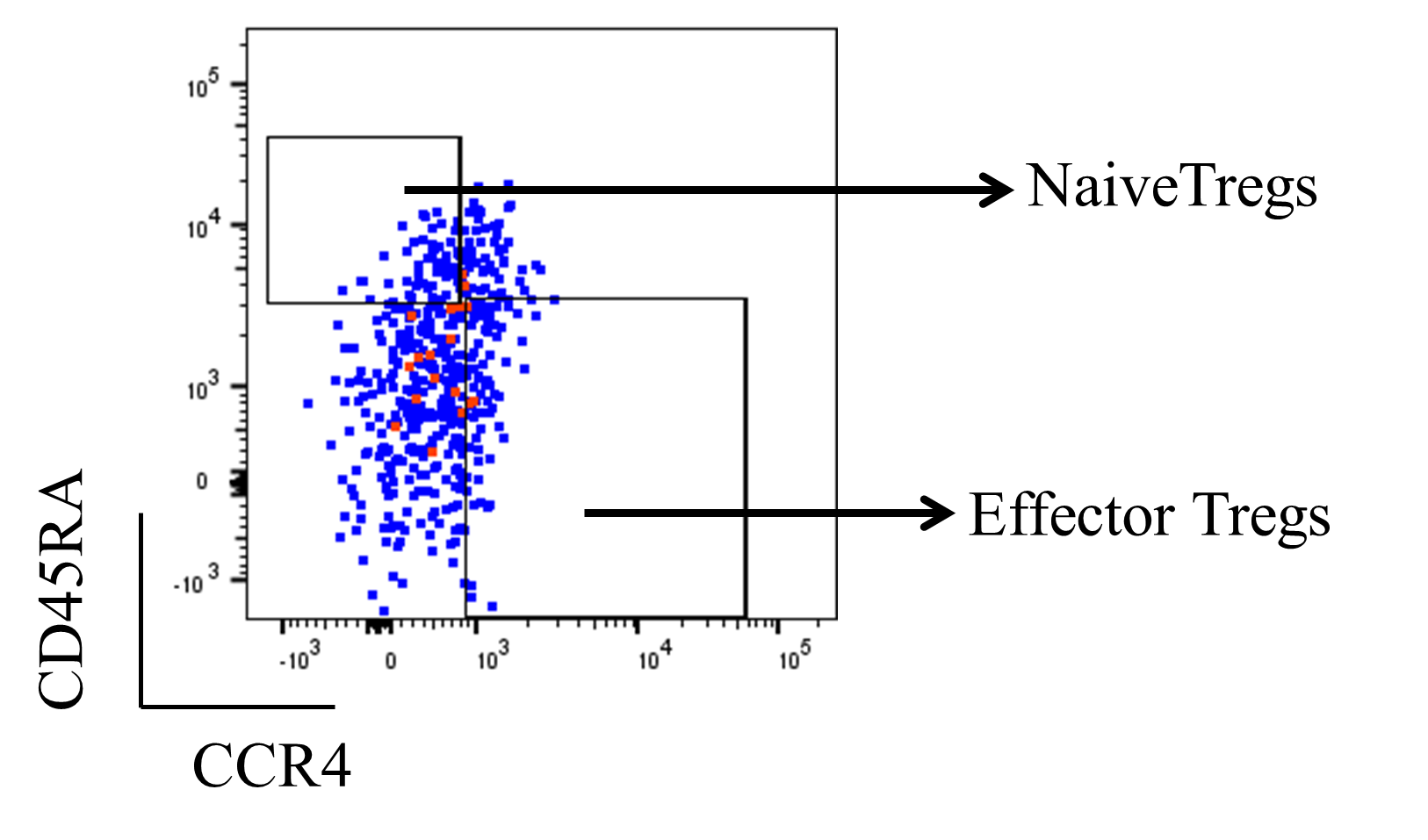


**Fig S3: Gating strategy for Treg subsets**

Tregs were gated as CD4^+^CD25^+^CD127^low/-^ cells as described in Fig S1. Mature naïve Tregs were further designated as CD45RA^+^ICOS^+/-^CD31^-^ Tregs, memory Tregs as CD45RA^-^ICOS^+/-^CD31^+/-^ Tregs, and recently thymus-emigrated Tregs as CD45RA^+^ICOS^+/-^CD31^+^ Tregs as described earlier [30].


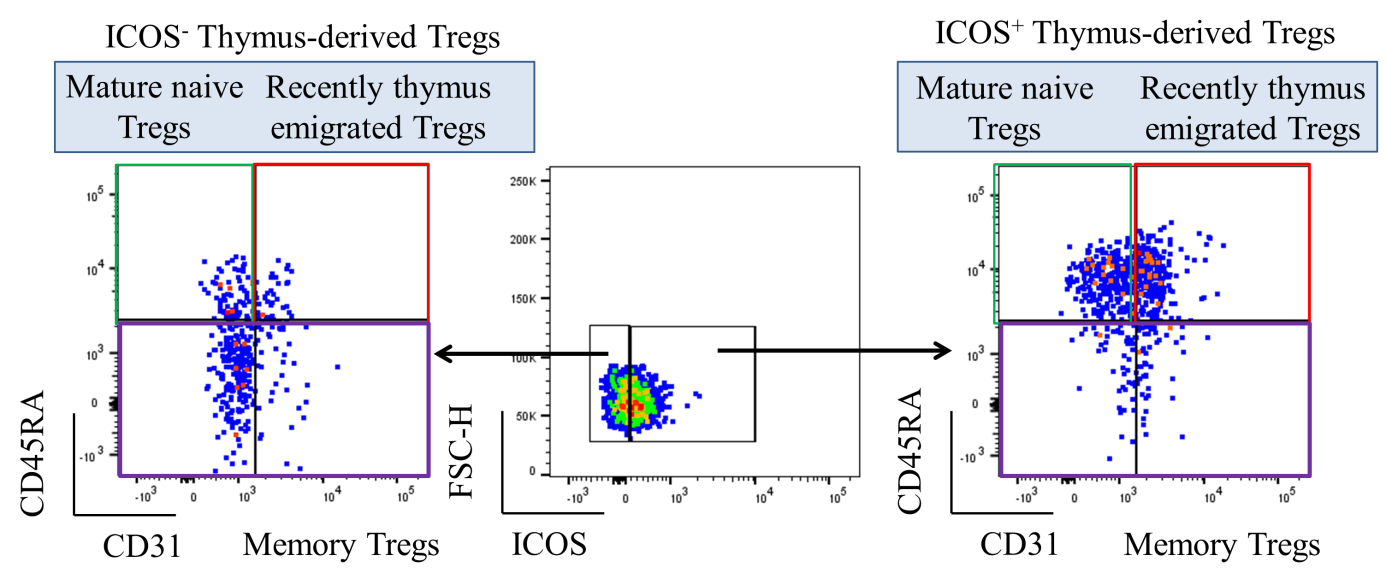


**Fig S4: Percentages of ICOS^+^ vs. ICOS^-^ mature naïve and memory Tregs.**

Tregs were gated as CD4^+^CD25^+^CD127^low/-^ cells as described in Fig. S1 and further gated as depicted in Fig. S3. (**a**) Memory Tregs were further designated as CD45RA^-^CCR4^+^ICOS^+/-^CD31^+/-^ Tregs and (**b**) mature naïve Tregs as CD45RA^+^CCR4^-^ICOS^+/-^CD31^+/-^ Tregs. Data were analyzed using a Student´s t-test or a one-way ANOVA test followed by a Tukey post-hoc test with * p-value <0.05, ** p-value <0.01, *** p-value <0.0001. Healthy controls are designed as CTRL (n=8), AD patients as AD (n=11) including patients without *FLG* null mutations as AD WT/WT (n=5) and patients with *FLG* null mutations as AD *FLG* MUT (n=6). AD patients were also stratified according to serum IgE levels (<100 kU/l, n=4; >100 kU/l, n=7) or according to EASI score (<5, n=7; >5, n=4).

**a**

**b**

**Fig S5: Percentages of ICOS^+^ vs. ICOS^-^ recently thymus-emigrated Tregs**

Tregs were gated as CD4^+^CD25^+^CD127^low/-^ cells as described in Fig S1 and further gated as depicted in Fig. S3. (**a**) Recently thymus-emigrated Tregs were further designated as CD45RA^-^ICOS^+/-^CD31^+^ Tregs. (**b**) Distribution of CD45RA^-^ICOS^+^CD31^+^ recently thymus-emigrated Tregs according to *FLG* mutation status, serum IgE levels or EASI score. (**c**) Distribution of CD45RA^-^ICOS^-^CD31^+^ recently thymus-emigrated Tregs according to *FLG* mutation status, serum IgE levels or EASI score. Data were analyzed using a Student´s t-test or a one-way ANOVA test followed by a Tukey post-hoc test with * p-value <0.05, ** p-value <0.01, *** p-value <0.0001. Healthy controls are designed as CTRL (n=8), AD patients as AD (n=11) including patients without *FLG* null mutations as AD WT/WT (n=5) and patients with *FLG* null mutations as AD *FLG* MUT (n=6). AD patients were also stratified according to serum IgE levels (<100 kU/l, n=4; >100 kU/l, n=7) or according to EASI score (<5, n=7; >5, n=4).

**a**

**b**

**c**

**Fig S6: Percentages of circulating Th-like Tregs.**

Tregs were gated as CD4^+^CD25^+^CD127^low/-^ cells as described in Fig. S1. Th2-like Tregs were designated as CD4^+^CD25^+^CD127^low/-^CCR4^+^CXCR3^-^CCR6^-^ cells, Th1-like Tregs as CD4^+^CD25^+^CD127^low/-^CCR4^-^CXCR3^+^ cells and Th17-like Tregs as CD4^+^CD25^+^CD127^low/-^CCR4^+^CXCR3^-^CCR6^+^CD161^+^ cells.
